# A prospective randomized clinical trial into the capacity of a toothpaste containing NovaMin to prevent white spot lesions and gingivitis during orthodontic treatment

**DOI:** 10.1186/s40510-015-0095-8

**Published:** 2015-08-13

**Authors:** Derek A. Hoffman, Andrew E. Clark, Wellington J. Rody, Susan P. McGorray, Timothy T. Wheeler

**Affiliations:** Department of Orthodontics, College of Dentistry, University of Florida, PO Box 100444, Gainesville, FL USA; 5555 Roanoke Trail, Tallahassee, FL 32312 USA; Department of Biostatistics, Colleges of Public Health and Health Professions and Medicine, University of Florida, PO Box 117450, Gainesville, FL USA

**Keywords:** White spot lesion, NovaMin, Orthodontics, Fluoride

## Abstract

**Background:**

White spot lesions and gingivitis represent common, yet challenging, dilemmas for orthodontists. Fluoride has shown some benefit as a protective measure against demineralization; however, this is usually insufficient for orthodontic patients with less than ideal oral hygiene. Dentifrices containing calcium sodium phosphosilicate bioactive glass (NovaMin) have been proposed to aid in prevention of white spot lesions and gingival inflammation. Thus, the purpose of this study was to determine if the use of NovaMin reduces the formation of white spot lesions and improves gingival health in orthodontic patients.

**Methods:**

This was a prospective, double-blind, randomized controlled trial. Forty-eight patients undergoing orthodontic treatment were randomly allocated to two groups. The control group consisted of 24 patients who received over-the-counter fluoride toothpaste (Crest®), while the study group consisted of 24 patients who were given the test dentifrice (ReNew™) containing 5 % NovaMin and fluoride. Patients were followed up for 6 months on a monthly basis. Decalcification, gingival health, plaque, and bacteria levels were evaluated every 3 months. Statistical analysis was performed using both parametric and non-parametric tests to identify differences between groups at different time points.

**Results:**

There were no significant differences between the groups in regard to changes in white spot lesions, plaque, or gingival health (*P* > 0.05). There was a trend toward improvement in white spot lesions found in subjects using Crest® at the 3-month time point; however, this was not sustained throughout the study.

**Conclusions:**

Our results indicate that a toothpaste containing NovaMin does not differ significantly compared to traditional fluoride toothpaste for improving white spot lesions and gingivitis in orthodontic patients.

## Background

White spot lesions (WSL) are the earliest macroscopic evidence of enamel caries [1] and represent a common, yet challenging, dilemma for orthodontists. Increased plaque retention leads to an increased microbial load which produces acid that lowers the pH. Subsequently, there is an increase in the porosity of the enamel allowing penetration of microorganisms to the subsurface layer that is hindered in its ability to remineralize. The majority of demineralization leading to WSL occurs in the subsurface region of the enamel [2]; thus, the outer 10 to 30 μm of enamel surface is believed to stay intact due to the supersaturation of fluorapatite [3]. In this scenario, calcium and phosphate ions have difficulty reaching the subsurface enamel layer to help remineralize [4]. Furthermore, salivary proteins known to inhibit demineralization, such as proline-rich proteins and statherin, are not able to pass through the enamel pores to protect this sublayer. As a result, this subsurface demineralization changes the refractive index of the enamel [5] and manifests clinically as a milky white opacity [6] or WSL.

Development of WSL during fixed appliance therapy can occur rapidly. Studies by both O’Reilly et al. [7] and Ogaard et al. [8] both show development of clinically visible WSL in orthodontic patients that occurred in 4 weeks or less. This approximates a minimal time interval between consecutive orthodontic appointments. WSL in orthodontic patients are typically found near the bracket base and usually have a crescent shape. They can also be detected under loose bands or as linear defects near the margin of the band [6]. Gorelick et al. [9] studied the incidence of WSL in orthodontic patients and found that almost 50 % of orthodontic patients developed at least one white spot lesion during the course of treatment. Maxillary lateral incisors showed the highest incidence of white spot lesions, followed by mandibular canines and first premolars.

The presence of WSL upon completion of orthodontic treatment is a major detractor from what would otherwise be a great esthetic result [10]. Both early detection and treatment can prove difficult at times, making prevention a critical component of managing this clinical problem. ReNew™ (Sultan Health Care, Englewood, NJ), a prescription-strength dentifrice containing 5 % calcium sodium phosphosilicate bioactive glass (NovaMin) and 5000 ppm neutral sodium fluoride, has been proposed to aid in prevention and reversal of white spot lesions. When immersed in an aqueous environment, sodium (Na) particles from the NovaMin begin to exchange with H^+^ ions. This allows for the release of calcium and phosphate from the calcium sodium phosphosilicate particles. The reaction of sodium ions with the hydrogen cations causes transient increase in pH that facilitates precipitation of calcium and phosphate from both the NovaMin and saliva to form a calcium phosphate layer on the tooth surface. These reactions and depositions continue until the depositions eventually crystallize into hydroxycarbonate apatite, which is structurally and chemically similar to biological hydroxyapatite [11]. It has also been proposed that a combination of fluoride and NovaMin is beneficial and synergistic in remineralization efforts. This is due to the ability of NovaMin to provide calcium and phosphorous ions in the production of fluorapatite [4, 12]. The availability of these ions is normally the limiting factor in fluoride treatment. Additionally, the transient increase in pH created by NovaMin can help resist demineralization.

NovaMin has also been shown to be beneficial in reducing gingivitis. A study by Tai et al. [13] showed improvement in gingival health over a 6-week period with the use of a dentifrice containing NovaMin. The exact mechanism of NovaMin’s antibacterial property remains unclear; however, it is proposed that the sodium and calcium content of the product affects bacterial liquid balance [14]. Therefore, the aim of this study was to determine if the use of a dentifrice containing calcium sodium phosphosilicate bioactive glass (NovaMin) reduces formation of white spot lesions and gingivitis in orthodontic patients.

## Methods

The study protocol was reviewed and approved by the Institutional Review Board at the University of Florida (Study # 329-2011). Patients receiving orthodontic care with full-fixed appliances in the graduate orthodontic clinic were selected according to the following criteria: (1) between the ages of 12 and 25, (2) moderate or poor oral hygiene, (3) good general health, (4) at least 6 months of orthodontic treatment remaining, (5) fixed orthodontic appliances present on all maxillary and mandibular anterior teeth, and (6) under the care of a general dentist at time of recruitment. Informed consent was obtained from the patient or the parent/legal guardian if under the age of 18. Patients with excellent oral hygiene, active dental caries, positive pregnancy test, or active periodontal disease were excluded from the study.

The sample size for this study was based on comparing the two groups for mean change in decalcification index (baseline to 6 months), and standard deviation estimates were from a previous study in a similar group of subjects undergoing orthodontic treatment. A two-sided two-sample *t*-test was used, with level of significance set at 0.05. With a sample size of 20 to 25 per group, we have a greater than 0.85 power to detect a difference of 1.0 unit change in the decalcification index (assuming a s.d. of 1.0) and a greater than 0.80 power to detect a difference of 1.25 if the s.d. is larger than anticipated (1.3).

This was a prospective, double-blind, randomized clinical trial. Subjects were initially screened and assigned to the control or experimental groups by means of block randomization. A total of 48 patients were enrolled in the study. Data was collected on 44 patients throughout the 6-month study period. Lack of data on the four subjects was due to possible allergic reaction in one patient and missed appointments by the other three. The control group encompassed 24 patients (15 males/9 females) with a mean age of 15.3 years who received commercial toothpaste containing 0.15 % fluoride (Crest®, Procter and Gamble, Cincinnati, OH). The experimental group consisted of 24 patients (17 males/7 females) with a mean age of 15.6 years who were given a toothpaste containing NovaMin (ReNew™, Sultan Healthcare, Englewood, NJ). All toothpaste tubes were covered with blinding labels and weighed before being dispensed to subjects. At the baseline appointment, patients received a professional dental cleaning, oral hygiene methods were reviewed, and photographs were taken to capture a baseline assessment of the decalcification index (DI). Subjects were instructed to bring the tube of toothpaste to each monthly appointment, at which time old toothpaste was collected, new toothpaste was dispensed, and oral hygiene instructions were reinforced. Toothpaste was also weighed after collection as a means to measure patient compliance.

At the 3- and 6-month follow-up appointments, pictures were taken, and the following clinical procedures were carried out in the maxillary and mandibular anterior teeth:

1. *Measurement of decalcification*: the DI used in the study was a modified version of the WSL index developed by Gorelick et al.[9]. The modified decalcification index scores individual teeth as follows: (0) no white spot lesion present, (1) visible white spots without surface interruption (mild decalcification), (2) visible white spot lesion having a roughened surface but not requiring a restoration (moderate decalcification), (3) visible white spot lesion with surface interruption (severe decalcification), and (4) cavitation. DI scoring was done with the photographs taken at the beginning of the study serving as documentation. The same camera settings were used throughout the study.
2. *Measurement of gingivitis*: we used the modified gingival index (GI), defined by Lobene et al. [15], as follows: (0) normal (no inflammation), (1) mild inflammation (slight change in color, little change in texture) of any portion of the gingival unit, (2) mild inflammation of the entire gingival unit, (3) moderate inflammation (moderate glazing, redness, edema, and/or hypertrophy) of the entire gingival unit, and (4) severe inflammation (marked redness and edema/hypertrophy, spontaneous bleeding or ulceration) of the gingival unit.
3. *Measurement of plaque*: the plaque index (PI) used in this study was the Turesky modification of the Quigley-Hein index [16]. Scoring used for this index is on a zero to five scale and is defined as (0) no plaque; (1) separate flecks or discontinuous bands of plaque at the gingival margin; (2) thin (up to 1 mm), continuous band of plaque at the gingival margin; (3) band of plaque wider than 1 mm but less than one third of the tooth surface; (4) plaque covering between one third and two thirds of the tooth surface; and (5) plaque covering more than two thirds of the tooth surface.
4. *Plaque bacterial counts*: relative *Streptococcus mutans* and *Lactobacillus* levels were measured in saliva using a commercial caries risk test (CRT-Bacteria, Ivoclar, Vivadent, Amherst, NY).

Inter-examiner reliability was assessed between two investigators (AEC and DAH) by scoring the indices of five randomly selected patients and time points from the study sample. Two-sample *t*-tests were used to test for differences between treatment groups, while paired tests were used to examine changes over time within treatment groups. Non-parametric tests were also used (Wilcoxon rank-sum and Wilcoxon signed-rank, respectively). While we expected outcome variables to be normally distributed, differences between parametric and non-parametric testing would alert us to cases where this may not be true. Results did not vary; thus, only the parametric results will be presented. A *P* value less than 0.05 was considered statistically significant. Chi-square tests, Fisher exact test, two sample *t*-tests, and Wilcoxon rank-sum tests were used to analyze any significant differences in regard to sex, race, age, and time in treatment between the control and experimental groups.

## Results

**Fig. 1:**
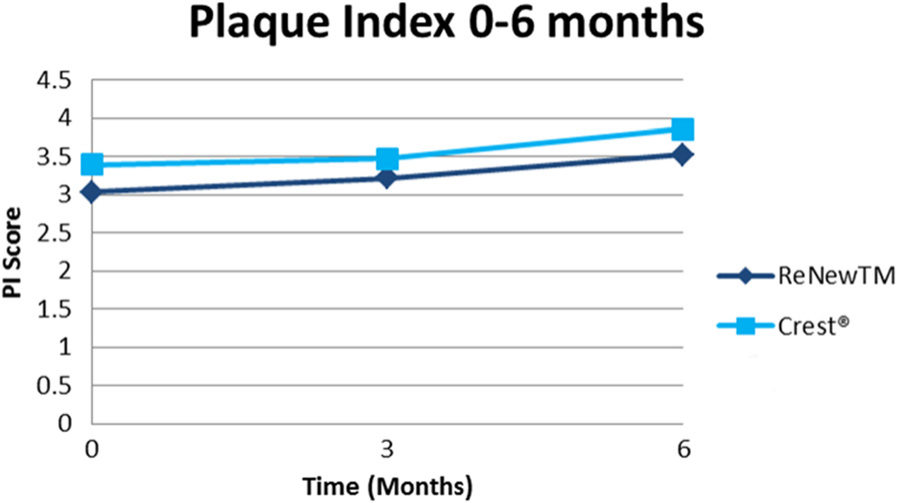
Mean plaque index (PI) score for each group throughout the study. No statistically significant difference was found at 3 and 6 months

**Fig. 2:**
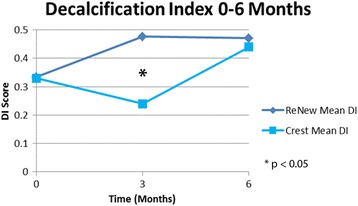
Mean decalcification index (DI) score for each group 0–6 months. *Statistically significant difference found at 3 months. This was not realized at 6 months

**Fig. 3:**
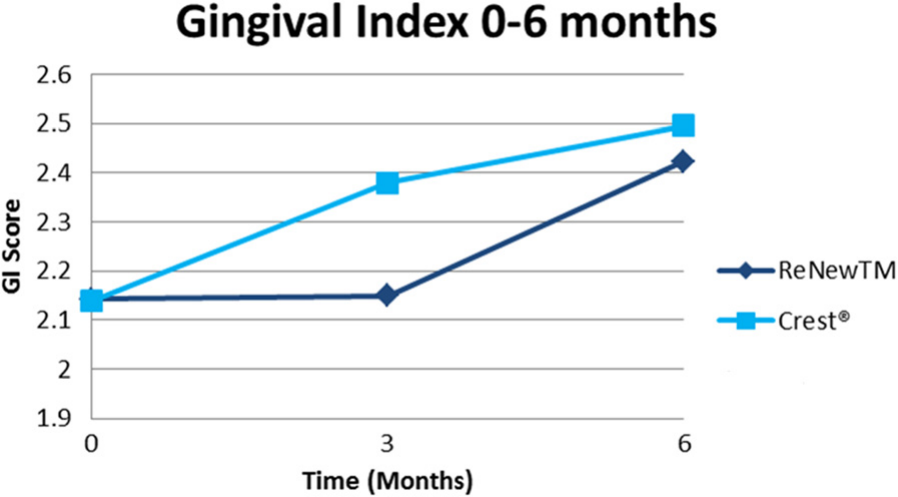
Mean gingival index (GI) score 0–6 months. No statistically significant difference found at 3 and 6 moths

Statistical analysis showed that the groups were similar regarding a number of variables at baseline, including age, time in treatment, DI, GI, and PI scores (Table 1). They also did not differ significantly with respect to sex (*P* = 0.54, chi-square test) or race (*P* = 0.72, Fisher exact test). No clinical trends or statistically significant difference between the control group (Crest®) and experimental group (ReNew™) was noted throughout the 6-month follow-up for PI (Fig. 1, Table 2). There was a trend toward improvement in white spot lesions (DI score) found in subjects using Crest® at the 3-month time point, which was statistically significant (*P* = 0.0403) (Fig. 2, Table 2). Likewise, the ReNew™ group showed a trend toward improvement in gingival health at the 3-month time point; however, no statistically significant difference was detected (Fig. 3, Table 2). These improvements were not sustained throughout the study since no statistically significant differences were detected between the control and treatment groups at the 6-month time point for all three indices (DI, GI, and PI).

**Table 1:** Baseline comparison between groups

	ReNew™	Crest®	*P* value (*t*-test)
Mean DI score	0.33	0.33	0.27
Mean GI score	2.14	2.15	0.95
Mean PI score	3.04	3.41	0.27
Mean age	15.6	15.3	0.63
Tx time	1.5	1.2	0.23

*P* value was set at 0.05. No significant difference between groups at baseline were detected using two-sample *t*-test

**Table 2:** Comparison at 3-month and 6-month intervals

	Decalcification index (DI)						
	Number	Mean	Median	St dev	Min	Max	*P* value (*t*-test)
Baseline ReNew	24	0.33	0.21	0.34	0	1.17	
Baseline crest	24	0.330	0.250	0.4	0	1.33	0.97
3 month ReNew	24	0.48	0.33	0.44	0	1.42	
3 month crest	23	0.24	0.17	0.3	0	1.250	0.0403*
6 month ReNew	23	0.47	0.42	0.37	0	1.58	
6 month crest	21	0.440	0.250	0.470	0	2.08	0.81
Plaque index (PI)							
Baseline ReNew	24	3.04	3.250	1.33	0	4.92	
Baseline crest	26	3.39	3.63	0.93	1.58	5	0.27
3 month ReNew	24	3.22	3.42	1.16	1.17	5	
3 month crest	23	3.48	3.33	0.9	1.750	4.83	0.41
6 month ReNew	23	3.53	3.42	1.09	1.92	5	
6 month crest	21	3.86	4.09	1.07	1	5	0.32
Gingival index (GI)							
Baseline ReNew	24	2.14	2.13	0.61	1.42	3.83	
Baseline crest	24	2.15	2.250	0.47	1.250	3	0.95
3 month ReNew	24	2.15	2.04	0.52	1.17	3	
3 month crest	22	2.35	2.250	0.43	1.67	3.17	0.17
6 month ReNew	23	2.42	2.33	0.560	1.33	3.42	
6 month crest	21	2.5	2.42	0.63	1.33	4	0.69

*P* value was set at 0.05 using two-sample *t*-test

*P < 0.05 indicating a significant difference between groups for DI at 3 months

We also examined change from baseline to 3 and 6 months, comparing the two groups. No statistically significant differences were detected. Within-group comparisons detected significant changes from baseline to 6 months for DI and PI in the experimental group (*P* = 0.0453, 0.0405, respectively), indicating significant worsening over time for these indices. The control group had similar changes for DI and PI, which were of borderline significance (*P* = 0.13, *P* = 0.09, respectively). A significant increase in GI from baseline to 6 months was detected for the control group (*P* = 0.0143), while a similar change of borderline significance (0.06) was observed for the treatment group.

No difference was found between groups in regard to compliance: 41.7 % of subjects in each group were non-compliant with the usage of dispensed toothpaste. Relative bacteria counts of *S. Mutans* and *Lactobacillus* were assessed between the ReNew™ and Crest® groups, and no statistically significant difference was found between treatment groups nor were differences detected in PI, GI, or DI changes over 6 months when low- and high-bacteria groups were compared (all *P* > 0.05).

## Discussion

Use of calcium and phosphorous to prevent and/or reverse white spot lesions shows promise in research studies [17–19]. There are many different delivery systems for calcium and phosphorous compounds. For the purpose of comparing our results with that of other research, we will consider all delivery vehicles to be equally effective. Thus, our results are in agreement with a study by Huang et al. [20] that showed no difference between MI Paste Plus, PreviDent mouthwash, and standard homecare regarding improvement of WSL over an 8-week period. Results are also in agreement with the in vitro study by Ballard et al. [21] who tested Restore (which contains NovaMin), PreviDent 5000, and MI Paste Plus. Results of this study showed none of these products were more effective at esthetically resolving white spot lesions than a control.

A study by Robertson et al. [17] revealed a statistically significant improvement in white spot lesions (WSL) using MI Paste Plus, as compared to a control (Tom’s of Maine toothpaste). In this study, 50 patients were followed for 3 months. Results showed a 53.5 % decrease in decalcification index (DI) scores in the MI Paste Plus group, while the control group showed a 91.1 % increase. Possible reasons for the difference in this study may be due to the scoring system used, which measured decalcification on a zero to three scale based on size and did not take into account surface roughness. This scoring system also divides the facial surface of the tooth into quadrants (mesial, distal, incisal, and gingival) increasing the amount of data points available, thereby making any difference easier to detect. Scoring was also assessed by means of photographs which may not be as clinically accurate and could possibly induce bias.

The role of fluoride in prevention of tooth decalcification is well documented [7, 22–24]. Nevertheless, there is debate regarding the amount of fluoride that should be prescribed when remineralization of subsurface enamel is desired [6, 25]. A limiting factor in gaining remineralization, or reversal, of white spot lesions during this study may have been the relatively high concentration of fluoride in the ReNew™ dentifrice. High concentrations of fluoride will lead to remineralization of the surface layer with fluorapatite, which may inhibit remineralization of subsurface layers [6]. This factor may help explain the improvement seen in the control group (Crest®) at the 3-month mark. Low levels of fluoride working in conjunction with calcium and phosphorous found in saliva may have led to a temporary improvement in white spot lesions. We speculate that this transient improvement was eventually overcome by the acidic attack from increasing plaque levels. It is also possible that this group may have shown a transient improvement in hygiene, and hence white spot lesions, for 1–2 months simply because they knew they were enrolled in a study evaluating this outcome.

One vehicle which may be beneficial in providing the calcium- and phosphorous-limiting remineralization is by encouraging patients to chew gum with casein phosphopeptide amorphous calcium phosphate (CPP-ACP). This in combination with fluoride levels found in over-the-counter toothpastes may encourage reversal of WSL in orthodontic patients. Another means to reverse WSL with CPP-ACP would be the application of MI Paste Plus, after application of phosphoric acid on the WSL for 30 s. Application of phosphoric acid has proven beneficial in removing surface proteins and fluorapatite for resin-infiltration procedures. Further research is warranted to elucidate clinically predictable methods to prevent or reverse white spot lesions, particularly in orthodontic patients.

Evidence-based dentistry has become a frequent issue in recent dental literature [26]. In this randomized clinical trial, we tried our best to exclude confounding factors that may have interfered with the association between WSL/gingivitis and dentifrice type. Nevertheless, we still faced limitations that are now discussed. The most important one is that plaque and gingival hyperplasia prevented a completely accurate assessment of WSL in some patients. After rinsing with a disclosing agent, patients were instructed to brush until all plaque was removed. However, some plaque typically remained especially in hard to reach areas. A scaler was used to remove any remaining plaque, although given clinical time restraints this was occasionally difficult to achieve. Patient compliance is another limitation of this study. Compliance, as measured by the weight of returned toothpaste appears equal between both groups. However, if a patient did return old tubes of toothpaste at monthly visits, the patient was considered non-compliant, when in reality he/she may have been compliant. A final limitation of this study may have been the DI used. This index is slightly subjective and rated white spot lesions on a zero to four scale where patients rarely reached a score of 2 or higher. Our patients typically showed WSL on only a couple of anterior teeth. This may have made detecting a true difference more difficult. Future studies may consider using fluorescence technology.

## Conclusions

Results of this study show there is no difference between an over-the-counter fluoride containing toothpaste (Crest®) versus a toothpaste containing NovaMin (ReNew™) in their ability to improve white spot lesions, plaque levels, and gingival health in orthodontic patients.
